# Gadoxetic acid-enhanced MRI for the detection of liver metastases from melanoma

**DOI:** 10.1371/journal.pone.0313212

**Published:** 2024-11-04

**Authors:** Hyun Jung Chung, Nieun Seo, Kyunghwa Han, Heejin Bae, Yong Eun Chung, Minkyu Jung, Mi-Suk Park

**Affiliations:** 1 Department of Radiology, Severance Hospital, Yonsei University College of Medicine, Seoul, Korea; 2 Department of Radiology, Yonsei Biomedical Research Institute, Research Institute of Radiological Science, Seoul, Korea; 3 Division of Medical Oncology, Department of Internal Medicine, Yonsei University College of Medicine, Seoul, Korea; Suryabinayak Municipal Hospital, NEPAL

## Abstract

**Purpose:**

We aimed to assess imaging findings and detection sensitivity for melanoma liver metastases on gadoxetic acid-enhanced magnetic resonance imaging (MRI).

**Methods:**

This retrospective study included patients with melanoma liver metastasis who underwent gadoxetic acid-enhanced MRI. Two abdominal radiologists independently evaluated signal characteristics of liver metastases on morphologic imaging (precontrast T1- and T2-weighted imaging), diffusion-weighted imaging (DWI), dynamic imaging, and hepatobiliary phase (HBP). Imaging findings were compared according to detection on HBP and the primary site of the melanoma using logistic regression with the generalized estimating equation (GEE). Detection sensitivity for metastases was compared among different MR imaging sets using logistic regression with GEE.

**Results:**

A total of 67 patients with 254 liver metastases were included (44 women; mean age ± standard deviation, 65.6 ± 13.0 years). On HBP, 76.0% of metastases were detected, and 55.5% (141/254) showed hypointensity. Most of the metastases that were not detected on HBP originated from ocular melanomas (98.4%, 60/61), ≤1 cm (90.2%, 55/61) and showed T1 hyperintensity (98.4%, 60/61). Metastases from non-ocular melanomas more frequently showed T1 hypointensity, T2 hyperintensity, diffusion restriction, arterial enhancement, and HBP hypointensity than those from ocular melanomas (*Ps* ≤ 0.019). The detection sensitivity of HBP (76.0%) was significantly higher than DWI (65.7%, *P* = 0.006), but lower than morphologic imaging (98.8%, *P* < 0.001) and dynamic imaging (97.6%, *P* < 0.001).

**Conclusion:**

The detection sensitivity of HBP for melanoma liver metastasis was 76.0%, which was lower than that of morphologic or dynamic imaging. HBP of gadoxetic acid-enhanced MRI has little advantage in detecting melanoma liver metastases.

## Introduction

Around 97.0% of melanomas have their primary location identified, which is most frequently the skin, followed by the eye and mucous membrane [1]. Despite the low death rate of melanoma, patients with metastatic melanoma have poor prognosis [2]. In its early stage, melanoma is most often curable simply with surgical removal of the primary tumor [3, 4]. However, in advanced stages, the 5-year survival rate of metastatic melanoma patients goes down to 5.0–19.0% [5]. Thus, for metastatic melanoma, accurate tumor detection and staging are critical to choosing the best treatment plan and predicting patient prognosis [3, 4].

The liver is the most common initial site of metastasis from melanoma [4, 6, 7]. Since patient prognosis heavily depends on liver involvement, it is important to evaluate liver metastasis accurately [7–10]. Magnetic resonance imaging (MRI) is already well known for its superiority over other modalities for liver metastases from other origin tumors such as colorectal cancer, particularly for small metastases less than 1 cm [11–15]. According to previous studies, MRI shows superior sensitivity and specificity for the characterization of hepatic metastases from melanoma over other modalities such as ultrasonography and computed tomography, thus gaining traction as an imaging tool for metastatic melanoma in recent years [7, 16–20].

There are two types of contrast agents used for liver MRI, the extracellular contrast agent (ECA) and the hepatobiliary agent (HBA) [12]. HBA such as gadoxetic acid offers an additional hepatobiliary phase (HBP) which enables the detection of non-functioning hepatocyte lesions in the background of strongly enhanced normal liver parenchyma [13, 21, 22]. Gadoxetic acid-enhanced MRI has shown excellent sensitivity, ranging from 89.9 to 96.3%, for colorectal liver metastases [11, 13]. However, there are specific considerations when assessing melanoma liver metastases on MRI. Melanoma liver metastases are often small in size and show high signal intensity (SI) on T1 weighted imaging (T1WI) due to the T1 shortening effect generated by the melanin pigment or extracellular met-hemoglobin from intratumoral hemorrhage [4, 17]. Because of these unique signal characteristics, some melanoma liver metastases are not well delineated on HBP, thus HBP might not be quite useful for detecting liver metastases from melanoma. However, the signal characteristics and potential additional value of HBP have rarely been studied for melanoma liver metastases.

Therefore, the purpose of this study was to evaluate the imaging findings and detection sensitivity of gadoxetic-enhanced MRI for melanoma liver metastases. In addition, we aimed to compare these MR findings according to the primary site of the melanoma.

## Materials and methods

### Study population

The Institutional Review Board of Severance Hospital approved this retrospective study and required neither patient approval nor informed consent for our review of patient images and medical records. The data were accessed for research purposes on April 27th, 2022, and the authors did not have access to information that could identify individual participants during or after data collection. From August 2005 to May 2021, patients with liver metastasis from melanoma who underwent liver MRI were consecutively enrolled. Among them, patients without gadoxetic acid-enhanced MRI, with other concurrent malignancies, or with prior systemic treatment were excluded. Patients with inadequate confirmation of liver lesions or imaging artifacts were also excluded. Clinical information including age, sex, pathology, and the primary site of the melanoma was extracted from electronic medical records.

### Acquisition of MRI

Liver MRI was performed using one of eight MR machines (one 1.5-T and seven 3.0-T machines; GE HealthCare, Siemens Healthineers, and Philips Healthcare). All scans were performed using a consistent protocol within a single hospital with randomly selected MR machines. Routine liver MRI sequences included dynamic T1WI, dual-echo spoiled gradient-echo T1-weighted in-phase and opposed-phase imaging, multi-shot and single-shot turbo spin-echo T2-weighted imaging (T2WI), and single-shot echo-planar diffusion-weighted imaging (DWI) with b values of 50, 400, and 800 sec/mm^2^. Before and after the injection of gadoxetate disodium (Primovist, Bayer Schering Pharma, Berlin, Germany) at a dose of 0.1 mL/kg (0.025 mmol/kg) followed by a 20-mL saline flush at an injection rate of 1 mL/sec, a dynamic fat-suppressed spoiled gradient-echo T1WI was acquired. The bolus-tracking approach was used to calculate the timing of the arterial phase, with an 18-second delay starting from the moment of aortic enhancement. The subsequent dynamic phases, which included the portal and transitional phases, were scanned at intervals of approximately 30 seconds; the time needed for each dynamic phase ranged from 16 to 22 seconds. The HBP was obtained 15–20 minutes after the injection of the contrast agent.

### Image analysis

A study coordinator (with 4 years of experience in liver MRI) identified melanoma liver metastases using medical records, histopathologic results, and imaging. The study coordinator chose up to five liver metastases per patient in descending order of size and measured the size of each lesion. Hepatic metastases were confirmed by histopathologic examination (either biopsy or surgery) or by imaging. On MRI, a liver lesion was considered a metastasis if it showed 1) moderate T2 hyperintensity and T1 hypointensity relative to the liver, with diffuse or peripheral enhancement, or 2) T1 hyperintensity and moderate T2 hyperintensity or hypointensity relative to the liver, regardless of enhancement [23]. For the liver lesions not confirmative for metastasis, follow-up imaging (≥ 12 months) was re-evaluated to determine findings for metastases or disease progression.

Two board-certified abdominal radiologists (dedicated to liver MRI with more than 5 to 10 years of experience) independently evaluated the MR findings of liver metastases. Discrepancies in radiologic assessments between the two readers were resolved after a consensus review. The readers were aware that patients had melanomas with hepatic metastases, but they were blinded to other clinical data and pathologic findings. The SI of the lesion was evaluated in comparison with the background normal liver. If the lesion showed heterogeneous SI, the SI was determined by its dominant SI. For example, isointense SI was defined when a lesion was isointense in most areas with smaller hyper- or hypointense portions compared to the SI of the background liver. If the whole lesion showed exactly the same SI as the surrounding liver, the lesion was considered ‘not detected’. Some MRI scans did not include arterial phase subtraction imaging, so subtraction imaging was evaluated when available. Positive diffusion restriction was considered for a lesion showing high SI on the highest ’b’ value imaging and corresponded low SI on the apparent diffusion coefficient map.

### Statistical analysis

Data were presented as numbers and percentages for categorical variables or means ± standard deviations or medians with interquartile ranges (IQRs) for quantitative variables. All statistical analyses were lesion-based because the sensitivity for lesion detection was the primary endpoint of our study. First, the MR findings of liver metastases were compared according to their detection on HBP and according to the primary site of the melanoma, respectively. When the sizes of liver metastases were compared as continuous values, regression with the generalized estimating equation (GEE) was used. Other MR findings classified as categorial variables were compared using logistic regression with GEE. We used GEE approach in consideration of clustered data, because many patients had multiple liver lesions, and multiple lesions within the same patient are not truly independent. For a few variables that could not be estimated by GEE due to sparse data, the Chi-square test or Fisher’s exact test was performed.

Second, detection sensitivity for metastases was compared among four different MR imaging sets (morphologic imaging, DWI, dynamic imaging, HBP) using logistic regression with GEE, considering the clustering of lesions within patients. Morphologic imaging included combined noncontrast T1WI and T2WI, whereas dynamic imaging included contrast-enhanced T1WI (arterial, portal, and transitional phases). Bonferroni correction was applied to adjust *P* values for multiple comparisons. Adjusted *P* values were presented by multiplying the number of comparisons. To compare detection sensitivity among imaging sets, subgroup analyses were performed according to the size of metastasis (≤1 cm vs. >1 cm), and the primary site of the melanoma (ocular vs. non-ocular).

Lastly, the interobserver agreement for MR interpretation between the two readers was evaluated using the bootstrap method to estimate the correlated κ coefficients, considering the clustering of lesions within patients: κ values < 0.20, poor agreement; 0.21–0.40, fair; 0.41–0.60, moderate; 0.61–0.80, good; and 0.81–1.00, excellent [24]. Statistical analyses were performed using R software, version 4.2.2 *(http://www.R-project.org)*. *P* < 0.05 was considered statistically significant.

## Results

### Patient characteristics

**Fig 1:**
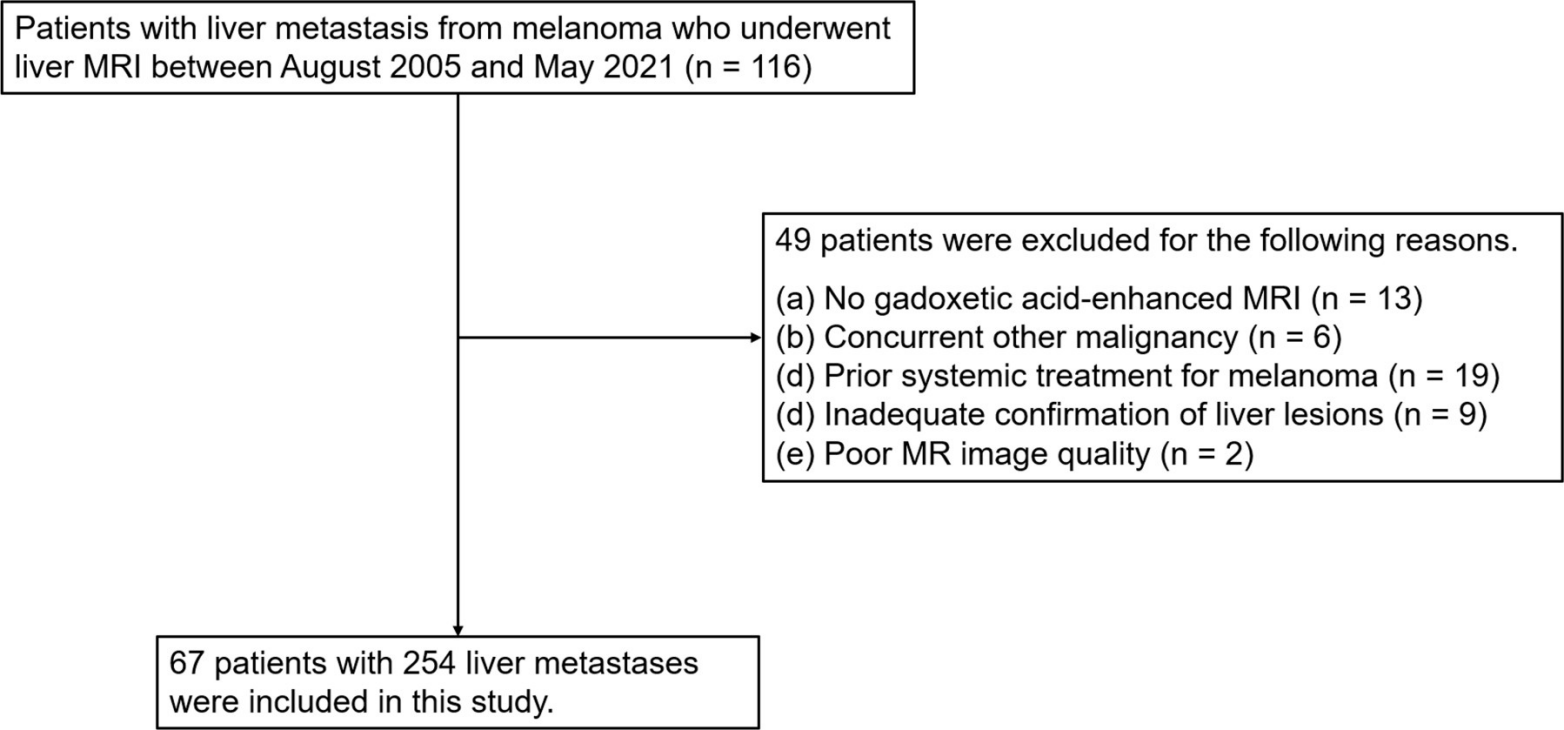
Patient flow diagram.

Among 116 patients with liver metastases from melanoma and liver MRI, 49 patients were excluded due to the following reasons: no gadoxetic acid-enhanced MRI (n = 13), concurrent other malignancy (n = 6), prior systemic treatment (n = 19), inadequate confirmation of liver lesions (n = 9) and poor MR image quality (n = 2). Finally, 67 patients with 254 liver metastases were included in this study (Fig 1; 44 women; mean age ± standard deviation [range], 65.6 ± 13.0 [28–88], years). Liver metastases were confirmed by pathology in 26 patients (22 with biopsy and 4 with surgical resection) and by imaging in 41 patients. Median number of liver metastases per patient was 5 (IQR, 2–5) (Table 1).

**Table 1:** Patient demographics.

Variables	Value
Age, year	
Mean ± standard deviation	65.6 ± 13.0 (28–88)
Sex	
Male	23 (34.3)
Female	44 (65.7)
Primary site	
Ocular	57 (85.1)
Liver	2 (3.0)
Skin	2 (3.0)
Rectum/Anus	2 (3.0)
Oro-nasal cavity	2 (3.0)
Esophagus	1 (1.5)
Vulva	1 (1.5)
Confirmation of metastasis	
Pathology	26 (38.8)
Imaging	41 (61.2)
Number of evaluated liver metastases per patient	
Median (Interquartile range)	5 (2–5)

Unless otherwise specified, data are numbers of patients with percentages in parentheses.

### Gadoxetic acid-enhanced MRI features of melanoma liver metastases

MR findings according to detection on HBP are summarized in Table 2. Among a total of 254 metastases, 86.2% (219/254) of lesions originated from ocular melanomas. The mean size of the metastases was 1.7 cm, and 43.7% (111/254) of them were ≤ 1 cm. Regarding all metastases, dominant SIs were high on T1WI (68.9%, 175/254) and high on T2WI (55.5%, 141/254), and 65.7% of lesions showed diffusion restriction. On dynamic phases, 80.3% of lesions showed hyperintensity on the arterial phase, and 56.1% of lesions showed arterial enhancement on subtraction imaging. Approximately half of the lesions showed hyperintensity on the portal (55.9%, 142/254) and transitional phases (48.4%, 123/254). To note, 24.0% (61/254) of lesions were not detected on HBP, and residual lesions showed hyperintensity (15.4%, 39/254), isointensity (5.1%, 13/254), or hypointensity (55.5%, 141/254) on HBP.

**Table 2:** MR findings of liver metastases according to the detection on the hepatobiliary phase.

	All (n = 254)	Detection (+) (n = 193)	Detection (-) (n = 61)	P-value
Primary site				0.002
Ocular	219 (86.2)	159 (82.4)	60 (98.4)	
Non-ocular	35 (13.8)	34 (17.6)	1 (1.6)	
Size (Mean ± SD), cm	1.7 ± 1.9	2.1 ± 2.1	0.6 ± 0.3	<0.001
Size				<0.001
≤1 cm	111 (43.7)	56 (29.0)	55 (90.2)	
>1 cm	143 (56.3)	137 (71.0)	6 (9.8)	
T1-weighted imaging				<0.001
Hypointense	58 (22.8)	57 (29.5)	1 (1.6)	
Isointense	3 (1.2)	3 (1.6)	0 (0)	
Hyperintense	175 (68.9)	115 (59.6)	60 (98.4)	
Not detected	18 (7.1)	18 (9.3)	0 (0)	
T2-weighted imaging				<0.001
Hypointense	41 (16.1)	33 (17.1)	8 (13.1)	
Isointense	1 (0.4)	1 (0.5)	0 (0)	
Hyperintense	141 (55.5)	139 (72.0)	2 (3.3)	
Not detected	71 (28.0)	20 (10.4)	51 (83.6)	
Diffusion restriction				<0.001
Yes	167 (65.7)	161 (83.4)	6 (9.8)	
No	87 (34.3)	32 (16.6)	55 (90.2)	
Arterial phase				<0.001
Hypointense	37 (14.6)	37 (19.2)	0 (0)	
Isointense	2 (0.8)	2 (1.0)	0 (0)	
Hyperintense	204 (80.3)	152 (78.8)	52 (85.2)	
Not detected	11 (4.3)	2 (1.0)	9 (14.8)	
Arterial phase subtraction^a^				<0.001
Enhancement	133 (56.1)	121 (68.4)	12 (20.0)	
No enhancement	104 (43.9)	56 (31.6)	48 (80.0)	
Portal phase				<0.001
Hypointense	74 (29.1)	72 (37.3)	2 (3.3)	
Isointense	12 (4.7)	12 (6.2)	0 (0)	
Hyperintense	142 (55.9)	98 (50.8)	44 (72.1)	
Not detected	26 (10.2)	11 (5.7)	15 (24.6)	
Transitional phase				<0.001
Hypointense	91 (35.8)	88 (45.6)	3 (4.9)	
Isointense	6 (2.4)	6 (3.1)	0 (0)	
Hyperintense	123 (48.4)	81 (42.0)	42 (68.9)	
Not detected	34 (13.4)	18 (9.3)	16 (26.2)	
Hepatobiliary phase				NA
Hypointense	141 (55.5)	141 (73.1)		
Isointense	13 (5.1)	13 (6.7)		
Hyperintense	39 (15.4)	39 (20.2)		
Not detected	61 (24.0)		61 (100)	

^a^A total of 237 lesions were evaluated on arterial subtraction imaging, because arterial subtraction imaging was not available for 17 lesions from 4 patients.

SD, standard deviation; NA, not applicable

Regarding MR findings according to detection on HBP, most of the undetected lesions on HBP were of ocular origin (98.4%, 60/61), and 90.2% (55/61) of the undetected lesions were ≤1 cm. Liver metastases detected on HBP showed the following MR findings more frequently than those not detected on HBP: low SI on T1WI, high SI on T2WI, diffusion restriction, arterial enhancement, and hypointensity on the portal and transitional phase (all *Ps* < 0.001). Notably, among 61 undetected lesions on HBP, 98.4% (60/61) showed hyperintensity on T1WI, and 90.2% (55/61) did not show diffusion restriction.

**Fig 2:**
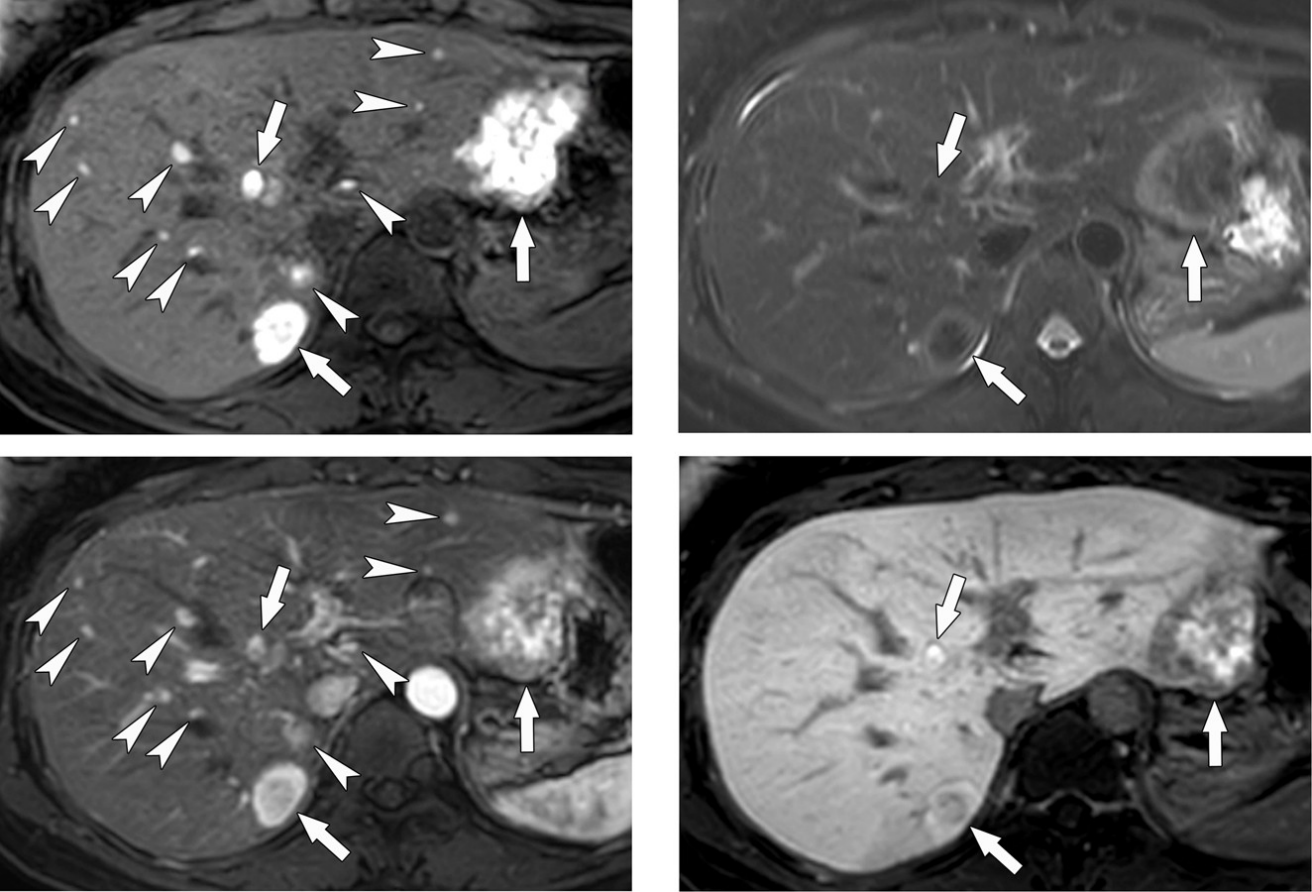
Gadoxetic acid-enhanced MRI in a 53-year-old woman with liver metastases from ocular (choroidal) melanoma. Axial gadoxetic acid-enhanced MR images show multiple hepatic metastases (arrows and arrowheads). Among these lesions, only three metastases are detected (arrows) on a fat-suppressed T2-weighted image (B), and the hepatobiliary phase (D). On precontrast (A) and arterial phase T1-weighted images (C), multiple hyperintense lesions are additionally detected in the liver, suggestive of metastases (arrowheads).

**Fig 3:**
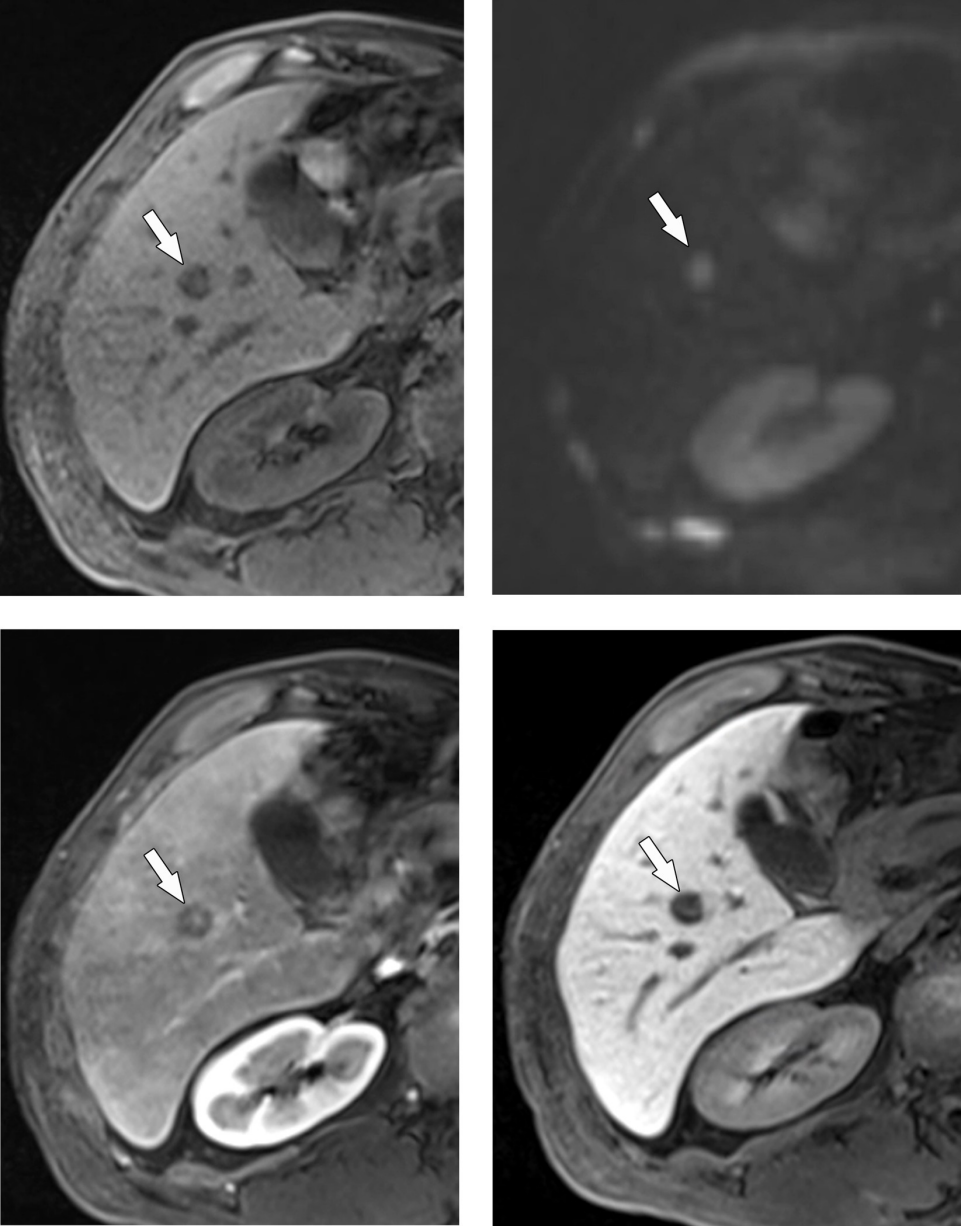
Gadoxetic acid-enhanced MRI in a 59-year-old man with a liver metastasis from non-ocular (oral mucosal) melanoma. Axial gadoxetic acid-enhanced MR images show a single metastasis in the right inferior liver (arrows). It shows low signal intensity on a precontrast T1-weighted image (A), high signal intensity on a diffusion-weighted image (b = 800 s/mm^2^) (B), low signal intensity with peripheral enhancement on a portal phase T1-weighted image (C), and low signal intensity on the hepatobiliary phase (D), compared to the surrounding liver.

MR findings according to the primary site of the melanoma are summarized in Table 3. Metastases of non-ocular origin showed the following MR findings more frequently than those of ocular origin: low SI on T1WI, high SI on T2WI, diffusion restriction, arterial enhancement on subtraction imaging, and low SI on HBP (*Ps* ≤ 0.019). Lesion size and SIs on the arterial, portal, and transitional phases did not significantly differ according to the primary site (*Ps* > 0.05). Representative cases are presented in Figs 2 and 3.

**Table 3:** MR findings of liver metastases according to the primary site of the melanoma.

	Ocular (n = 219)	Non-ocular (n = 35)	P-value
Size (Mean ± SD), cm	1.7 ± 1.7	2.2 ± 2.9	0.182
Size			0.242
≤1 cm	99 (45.2)	12 (34.3)	
>1 cm	120 (54.8)	23 (65.7)	
T1-weighted imaging			<0.001
Hypointense	46 (21.0)	12 (34.3)	
Isointense	3 (1.4)	0 (0)	
Hyperintense	158 (72.1)	17 (48.6)	
Not detected	12 (5.5)	6 (17.1)	
T2-weighted imaging			<0.001
Hypointense	38 (17.4)	3 (8.6)	
Isointense	1 (0.5)	0 (0)	
Hyperintense	110 (50.2)	31 (88.6)	
Not detected	70 (32.0)	1 (2.9)	
Diffusion restriction			0.019
Yes	135 (61.6)	32 (91.4)	
No	84 (38.4)	3 (8.6)	
Arterial phase			0.126
Hypointense	28 (12.8)	9 (25.7)	
Isointense	2 (0.9)	0 (0)	
Hyperintense	178 (81.3)	26 (74.3)	
Not detected	11 (5.0)	0 (0)	
Arterial phase subtraction^a^			<0.001
Enhancement	103 (51.0)	30 (85.7)	
No enhancement	99 (49.0)	5 (14.3)	
Portal phase			0.450
Hypointense	58 (26.5)	16 (45.7)	
Isointense	9 (4.1)	3 (8.6)	
Hyperintense	130 (59.4)	12 (34.3)	
Not detected	22 (10.0)	4 (11.4)	
Transitional phase			0.432
Hypointense	72 (32.9)	19 (54.3)	
Isointense	5 (2.3)	1 (2.9)	
Hyperintense	115 (52.5)	8 (22.9)	
Not detected	27 (12.3)	7 (20.0)	
Hepatobiliary phase			<0.001
Hypointense	112 (51.1)	29 (82.9)	
Isointense	13 (5.9)	0 (0)	
Hyperintense	34 (15.5)	5 (14.3)	
Not detected	60 (27.4)	1 (2.9)	

^a^A total of 237 lesions were evaluated on arterial subtraction imaging, because arterial subtraction imaging was not available for 17 lesions from 4 patients.

SD, standard deviation

### Comparison of detection sensitivity for liver metastasis among different MR sequences

Detection sensitivity was compared among four different MR imaging sets (morphologic imaging, DWI, dynamic imaging, and HBP), with the results presented in Table 4. For all metastases, the detection sensitivity of HBP (76.0%) was significantly lower than morphologic (98.8%, *P* < 0.001), and dynamic imaging (97.6%, *P* < 0.001). On the other hand, the detection rate of HBP was significantly higher than that of DWI (65.7%, *P* = 0.006). When compared with other imaging sets, no lesion was additionally detected on HBP.

**Table 4:** Comparison of detection sensitivity of MR imaging sets according to lesion size and primary site.

	Detection sensitivity				P-value^a^		
	Morphologic	DWI	Dynamic	HBP	Morphologic vs. HBP	DWI vs. HBP	Dynamic vs. HBP
All lesions (n = 254)	251 (98.8)	167 (65.7)	248 (97.6)	193 (76.0)	<0.001	0.006	<0.001
Lesion size							
≤1 cm (n = 111)	109 (98.2)	43 (38.7)	105 (94.6)	56 (50.5)	<0.001	0.102	<0.001
>1 cm (n = 143)	142 (99.3)	124 (86.7)	143 (100.0)	137 (95.8)	0.300	0.096	0.054
P-value^b^	0.144	<0.001	0.694	<0.001			
Primary site							
Ocular (n = 219)	216 (98.6)	135 (61.6)	213 (97.3)	159 (72.6)	<0.001	0.012	<0.001
Non-ocular (n = 35)	35 (100.0)	32 (91.4)	35 (100.0)	34 (97.1)	>0.999	0.738	>0.999
P-value^c^	0.235	<0.001	0.104	<0.001			

Data are numbers of detected lesions with percentages in parentheses.

^a^P values are obtained by comparing the detection sensitivity of HBP and each imaging set. These are adjusted P values using Bonferroni correction.

^b^P values are obtained by comparing the detection sensitivity of each imaging set according to lesion size.

^c^P values are obtained by comparing the detection sensitivity of each imaging set according to the primary site of the melanoma.

^‘^Morphologic’ indicates combined T1WI and T2WI, and ‘Dynamic’ indicates a combination of arterial, portal, and transitional phases.

T1WI, T1-weighted imaging; T2WI, T2-weighted imaging; DWI, diffusion-weighted imaging; HBP, hepatobiliary phase

In the subgroup analysis according to lesion size, metastases larger than 1 cm showed better detection sensitivity than smaller lesions on DWI (86.7% vs. 38.7%, *P* < 0.001) and HBP (95.8% vs. 50.5%, *P* < 0.001), but the detection sensitivity according to lesion size was not significantly different on morphologic (*P* = 0.144), and dynamic imaging (*P =* 0.694). For metastases larger than 1 cm, the detection sensitivity did not significantly differ between HBP and other imaging sets (*Ps* > 0.05). For smaller metastases (≤ 1 cm), the detection sensitivity of HBP (50.5%) was significantly lower than morphologic (98.2%, *P* < 0.001) and dynamic imaging (94.6%, *P* < 0.001).

Regarding the primary site of the melanoma, the detection sensitivity for metastases from ocular melanomas was significantly lower than those from non-ocular melanomas on DWI (61.6% vs. 91.4%, *P* < 0.001) and HBP (72.6% vs. 97.1%, *P* < 0.001). For metastases from ocular melanomas, the detection sensitivity among the four MR imaging sets showed similar trends to the results found with all metastases: the sensitivity of HBP (72.6%) was significantly lower than morphologic (98.6%, *P* < 0.001) and dynamic imaging (97.3%, *P* < 0.001), but was significantly higher than DWI (61.6%, *P* = 0.012). On the other hand, for metastases from non-ocular melanomas, the detection sensitivity did not significantly differ among the four imaging sets (*Ps* > 0.05).

Interobserver agreement for MR interpretation is summarized in Table 5. The interobserver agreement was lowest for arterial subtraction imaging (κ = 0.500). HBP showed good interobserver agreement (κ = 0.712). For the other sequences, overall interobserver agreement was good to excellent (κ, 0.684–0.868).

**Table 5:** Inter-reader agreement of the MRI evaluation.

MR Sequence	κ statistics (95% confidence interval)
T1-weighted imaging	0.743 (0.622–0.863)
T2-weighted imaging	0.734 (0.655–0.813)
Diffusion-weighted imaging	0.868 (0.784–0.952)
Arterial phase	0.684 (0.533–0.835)
Arterial phase subtraction	0.500 (0.355–0.646)
Portal phase	0.753 (0.676–0.830)
Transitional phase	0.772 (0.691–0.852)
Hepatobiliary phase	0.712 (0.643–0.780)

## Discussion

It is generally assumed that the use of liver-specific contrast agents for liver MRI can improve the detection of liver metastasis due to the high lesion-to-liver contrast on HBP [11, 12]. However, little research has been conducted on whether this also applies to the liver metastasis of melanoma, and especially lesions containing melanin. Our study concentrated on the gadoxetic acid-enhanced MRI findings of liver metastases from melanoma, focusing on the usefulness of HBP. On HBP, 55.5% of liver metastases showed hypointensity, 20.5% showed iso- to hyperintensity, and 24.0% were not detected. The metastases which could be detected on HBP were more frequently of non-ocular origin, larger in size, and showed T1 hypointensity, T2 hyperintensity, diffusion restriction, and arterial enhancement compared to those not detected on HBP. The overall detection rate of HBP was significantly higher than DWI, but lower than morphologic (combined T1WI and T2WI) or dynamic imaging.

Hepatic metastases from other origins typically show rim enhancement on early post-contrast phases, with incomplete centripetal progression on the portal venous and delayed phases [25]. On HBP, most liver metastases from malignancies other than melanoma typically demonstrate global or peripheral hypointense SI as they do not have functional hepatocytes [13]. However, the high T1 SI of melanoma liver metastases, which is frequently observed for metastases from pigmented uveal melanoma [23, 26], can preclude the detection of metastasis on HBP. Our study revealed that most undetected metastases on HBP (60/61) were hyperintense on T1WI. Remarkably, no lesion was additionally detected on HBP compared to other sequences. Generally, ECA offers superior quality of arterial phase than HBA, mainly due to frequent respiratory motion artifacts and a lower gadolinium dose of HBA. In addition, scan times are possibly longer with HBA, since the final post-contrast phase is obtained approximately 15 to 20 minutes after HBA injection [12]. Based on our results, HBA may not be preferred to ECA for detecting liver metastasis from melanoma.

DWI revealed the lowest detection sensitivity (65.7%) for melanoma liver metastasis among MR sequences, although its detection sensitivity was relatively higher for larger metastases and metastases from non-ocular melanomas. A previous study reported a sensitivity of 53.0–59.0% for DWI when detecting uveal melanoma liver metastasis and also suggested that DWI did not provide additional benefits to morphologic-dynamic imaging for the detection of melanoma liver metastasis [23]. This discrepancy in the performance of DWI for melanoma liver metastasis and other metastases such as colorectal/or neuroendocrine liver metastasis might be related to how the melanin component affects T2WI and DWI [23]. Notably, noncontrast morphologic imaging (T1WI and T2WI) showed excellent detection sensitivity, which is higher than DWI and HBP in our study. A previous study comparing the detection rate of MRI and positron emission tomography also showed perfect sensitivity when unenhanced T1WI and T2WI was used to detect melanoma liver metastasis [18]. These results are encouraging because noncontrast liver MRI can be used as a surveillance or diagnosis tool for melanoma liver metastasis.

In this study, MR findings of liver metastases were compared according to the primary site of the melanoma. Liver metastases from non-ocular melanomas showed less T1 hyperintensity, and more T2 hyperintensity, diffusion restriction, arterial enhancement and HBP hypointensity than metastases from ocular melanomas. Therefore, MR features of liver metastases from non-ocular melanomas are more similar to the typical appearance of liver metastases other than melanoma, and 97.1% of metastases from non-ocular melanomas were detected on HBP. These differences are probably because ocular melanomas are more commonly melanotic than non-ocular melanomas such as cutaneous melanomas [27]. Therefore, special consideration based on the knowledge of unique MR features is needed to interpret liver MRI for ocular melanoma metastases. However, most metastases included in this study originated from ocular melanomas, thus further studies including large number of metastases from non-ocular melanomas are warranted to generalize our results.

There are several limitations to this study. First, data were collected retrospectively, which may have inevitably resulted in a selection bias. Additionally, since we did not perform a sample size calculation in this retrospective study, caution is needed before interpreting it as a confirmatory study. Second, multiple different MRI machines were used over a long study period, which was unavoidable due to the low prevalence of melanoma and retrospective study design. Nonetheless, all scans were performed using a consistent protocol within a single hospital, and the MRI machines used for each examination were randomly selected, which could have minimized the impact of using different machines on the reliability of the results. Third, not all metastases were confirmed by pathology. However, a complete pathological evaluation of melanoma liver metastasis is clinically impossible, and we believe that the MRI characteristics and follow-up imaging might be sufficient for the diagnosis of metastases. Fourth, each MR sequence was not reviewed separately, thus interpretation of each sequence might be influenced by the findings of another sequence. Although we chose this study design to comprehensively evaluate the lesion-by-lesion signal characteristics of melanoma liver metastasis on gadoxetic acid-enhanced MRI, the detection rate of each sequence might be overestimated. Fifth, inter-reader agreement was relatively low in the arterial phase and arterial phase subtraction images. Weak arterial enhancement and respiratory motion artifacts of the arterial phase in gadoxetic acid-enhanced MRI, and possible misregistration artifacts in subtraction images might have resulted in lower inter-reader agreement. Additional efforts, such as optimizing image acquisition, minimizing transient severe motion, and further training for readers might help improve inter-reader agreement. Lastly, only two readers assessed MR findings in this study. As both readers were experts in liver MRI, the possibility of the detection sensitivity being overestimated cannot be ignored, and this should be considered when interpreting our study results. Future research with more readers from various backgrounds with different levels of experience might confirm the reliability and generalizability of our study results.

In conclusion, the detection sensitivity of HBP for melanoma liver metastases was 76.0%, which was lower than that of morphologic or dynamic imaging. HBP of gadoxetic acid-enhanced MRI has little value for detecting melanoma liver metastases, and particularly less for metastases from ocular melanomas. Based on our study results, future research could be conducted to establish optimal MRI protocols for detecting liver metastasis originating from melanomas, including the appropriate type of contrast agent and MR sequence, which may differ depending on the primary site of the melanoma.
